# General Practitioners Records Are Epidemiological Predictors of Comorbidities: An Analytical Cross-Sectional 10-Year Retrospective Study

**DOI:** 10.3390/jcm7080184

**Published:** 2018-07-27

**Authors:** Pierpaolo Cavallo, Sergio Pagano, Mario De Santis, Enrico Capobianco

**Affiliations:** 1 Department of Physics “E.R. Caianiello”, University of Salerno, Via Giovanni Paolo II, 132, 84084 Fisciano (SA), Italy; spagano@unisa.it; 2 Cooperativa Medi-Service, Via R. Lettieri, 2, 84129 Salerno, Italy; mariodesantis@osservatoriosanitario.it; 3 Miller School of Medicine, University of Miami, Gables One Tower, 1320 S Dixie Hwy, Miami, FL 33146-2930, USA; ecapobianco@med.miami.edu

**Keywords:** comorbidity, electronic health records, general practitioner, network analysis, general population, diabetes

## Abstract

Background. Comorbidity represents the co-occurrence of pathological conditions in the same individual, and presents with very complex patterns. In most cases, reference data for the study of various types of comorbidities linked to complex diseases are those of hospitalized patients. Such patients may likely require cure due to acute conditions. We consider the emerging role of EHR (Electronic Healthcare Records), and study comorbidity patterns in a general population, focusing on diabetic and non-diabetic patients. Methods. We propose a cross-sectional 10-year retrospective study of 14,958 patients and 1,728,736 prescriptions obtained from family doctors, and thus refer to these data as General Practitioner Records (GPR). We then choose networks as the tools to analyze the diabetes comorbidity patterns, distinguished by both prescription type and main patient characteristics (age, gender). Results. As expected, comorbidity increases with patients’ age, and the network representations allow the assessment of associations between morbidity groups. The specific morbidities present in the diabetic population justify the higher comorbidity patterns observed in the target group compared to the non-diabetic population. Conclusions. GPR are usually combined with other data types in EHR studies, but we have shown that prescription data have value as standalone predictive tools, useful to anticipate trends observed at epidemiological level on large populations. This study is thus relevant to policy makers seeking inference tools for an efficient use of massive administrative database resources, and suggests a strategy for detecting comorbidities and investigating their evolution.

## 1. Introduction

Frequently, the diagnoses that physicians find in examined patients refer to multiple diseases, possibly interdependently defined [1] and organized [2] based on symptoms (subjective data), signs (objective data, clinically evidenced) and diagnostic tools (objective data, instrumentally evidenced). Diagnoses with such outcomes identify a condition commonly called comorbidity [3].

Comorbidity has become a major health and societal burden [4,5]. The incidence of chronic/multi-morbid diseases in the presence of aging populations, is steadily growing [6]. For instance, 80% of the US health budget [7] is spent on patients with four or more diseases. Electronic Health Records (EHR) can provide useful comorbidity information [8], although they present a main inherent limitation: EHR outsourced by hospitals refer only to subjects with severe disease conditions that usually require specialized intervention. Conversely, General Practitioners (GP) have routinely access to a much larger population [9,10], not necessarily suffering from an acute pathological condition. Hence, the EHR spectrum covered by GPs, say GP records or GPR, is valuable because complementary, wider and more geo-localized than hospitalized patient EHR (also considering that an overlap is determined by appointments with specialists administered in most cases by family doctors). In Italy, GPR report clinically relevant information on population health, including the prescriptions administered persistently and not only at hospital discharge time. Therefore, GP prescriptions (GPP) can be considered a specific subtype of GPR, and are routinely collected by Italian Health Authorities for administrative purposes. The scope of this study is to assess the validity of GPP as an instrument for the analysis of comorbidities. Since the study is currently expanding beyond Salerno’s province, our preliminary results refer currently to Salerno’s area.

To properly address this question, we rely on: (a) Data from a specific geographic location regarding GPs and their patients, and (b) Analytic tools, in particular network analysis to examine the complex multidimensional relationships between variables of relevance to people’s health. The mathematical aspects of Network Theory [11] are beyond the scope of our work. In the present research we have used it as a visual analytics tool which could be used to find unexpected connections, which may lead to research hypotheses. The context of diseases is rarely a straightforward consequence of a genetic abnormality [12], rather the result of interaction between molecular processes and environmental cues and assembling information at genome, metabolome, proteome, neuroendocrine and immune systems scales, plus social, environmental and healthcare variables [13,14,15]. Interactome-driven diseasome models [16] have been proposed: they are network representations which integrate all known phenotype and disease gene associations, showing the relevant importance of some genes. Similarly, correlations between the symptoms of diseases may be related to the number of shared genetic associations [17].

An aspect to emphasize is that the approach proposed here can become very useful in light of the role of Big Data for the Public Health sector [18]. Similar to the systems epidemiology approaches commonly used for analysis, monitoring and forecasting of the effects of healthcare programs implementation (see for instance [18]), our goal is to approach comorbidity from GPP taking advantage of a few valid comorbidity indices [19,20]. Among the first applications, Medicare claims data for acute cardiovascular hospitalization have been successfully used for understanding and measuring comorbid conditions [21]. Our approach is particularly but not exclusively focused on the diabetes group of patients, and represents a scalable and generalizable analytical proposal supporting the decision processes of policymakers in the Public Health sector.

## 2. Experimental Section

This cross-sectional study concerns a secondary analysis of the GPP of a population of 14,958 patients living in the Salerno area (Italy). In particular, the GPP refer to the 2002–2013 time interval. The patients have been grouped by gender and age, separated in decades, starting from 15 (e.g., 15–25; 25–35 etc.) with the last group corresponding to age above 85.

This study has been approved by Ethical Committee Campania Sud (ref. 59 of 8 June 2016), and the data used, consisting of: anonymized patient ID, age, sex, date of prescription, prescription type, prescription code, and diagnosis code, have been managed according to the Declaration of Helsinki guidelines. The prescription type refers to drug, laboratory testing, etc., and the prescription code refers to the prescribed item and the diagnosis code refers to the relevant ICD9CM [22].

The ICD9CM is still the standard epidemiological tool used in Italy, even if a further version (ICD10) has been published; the diagnostic codes have been grouped into specific epidemiological areas corresponding to the 20 main groups of the ICD9CM classification; this classification is resumed, with examples, in Supplementary Materials, Table S1.

The records, each representing one single prescription, have been further analyzed for possible inconsistencies (in less than 0.1% of cases). The specific prescription rules for GPs in Italy are briefly recalled in the Supplementary Materials. The total number of analyzed GPP was 1,728,736; Table 1 reports their subdivision by type.

**Figure 1:**
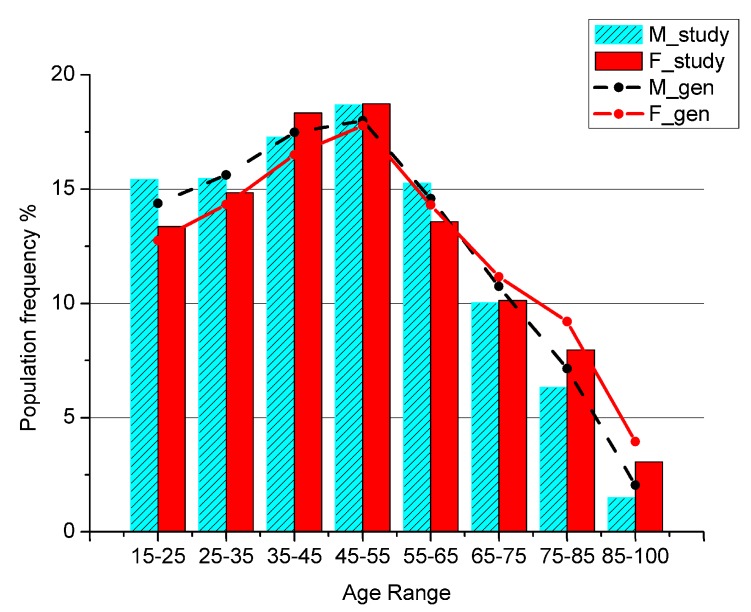
Percentage of men and women in the general population (M_gen, F_gen) and in the study sample (M_study, F_study) by age class.

To test for the representativeness of the sample with respect to the general population, the patients’ age distribution has been compared with the general population living in the area of research (Figure 1), referred to the last Italian Census data available.

The ages below 15 are not shown because in Italian Healthcare System the primary assistance for children is not covered by GPs, but only by Family Pediatricians, so a child is usually never visited by a GP.

The distribution of the ICD9CM diagnostic groups obtained from GPP, alone and separated by prescription type, was computed. To build a comorbidity network representation, each ICD9CM group was considered as a network node. A link is generated between two nodes if the corresponding ICD9CM codes are present in the same patient’s prescription. The average interval time between visits ranged from 20 to 70 days, depending on the patient’s age, with diabetic patients showing a higher frequency, ranging from 17 to 40 days; the frequency of close visits, within 3 days, was less than 5%.

Namely, if a patient receives prescriptions with reference to the diagnosis codes 401 = “essential hypertension”, belonging to the CIRC group (Circulatory Diseases) and 250 = “diabetes”, belonging to the META group (Metabolic Disease), a link is generated between the groups CIRC and META. A weight is associated to each node to count the number of times the specific ICD9CM group has been indicated, and a weight is associated to each link to count the number of times the corresponding ICD9CM groups have been co-prescribed. Finally, a strength was associated to each node as the sum of weights from all links.

Normalization was also performed: to compare networks obtained from different subsets of the original dataset, the values of the link weights, node weights, and strengths were divided by the number of patients present in each subset. The resulting networks are weighted networks with no self-loops, and have been computed using the R environment [23], using the package iGraph [24].

The resulting networks, obtained connecting ICD9CM groups through GPP information, show patterns specific to patients’ conditions. We specialized the analyses to comorbidity networks using GPP specific to diabetic (D) versus non-diabetic (ND) patients. The male gender was selected, because it receives no protective effect of sexual hormones up to menopausal age, thus it was expected not to find a worsening in the comorbidity condition in the age groups over 55. The subjects older than age 35 were studied, and two main groups were formed from the ICD9CM code 250: D and ND. The comorbidity networks were built from data of six age intervals: 35–45; 45–55; 55–65; 65–75; 75–85; above 85.

**Figure 2:**
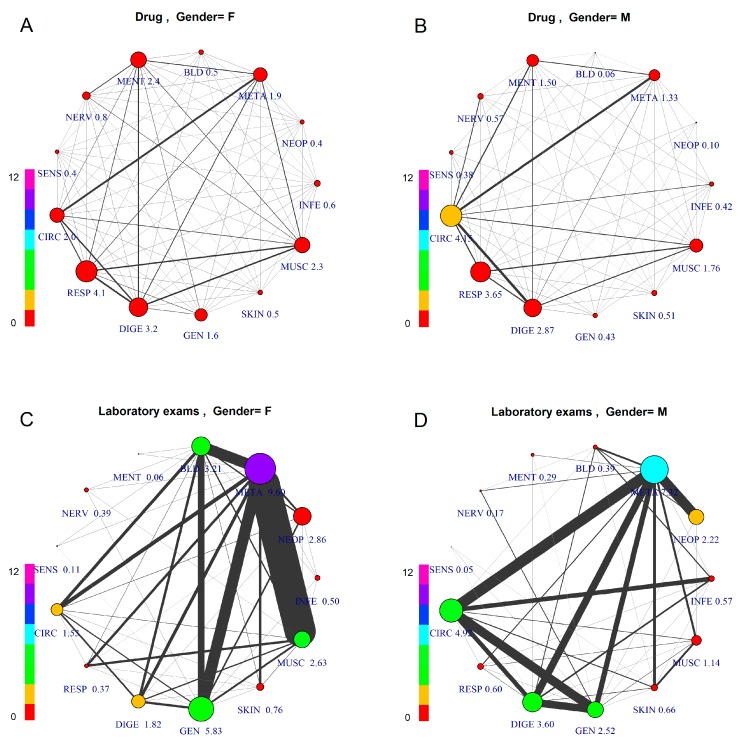
Comorbidity Networks by Patient Gender and Prescription Type. The subfigures represent patients aged 45–55, differentiated by gender and prescription type: (**A**) Female, Drugs; (**B**) Male, Drugs; (**C**) Female, Laboratory Tests; (**D**) Male, Laboratory Tests. The node size is proportional to the number of GPP per patient, with numerical value assigned to each specific label. The node color indicates overall number of co-prescriptions per patient, according to the scale at the left side.

The following aspects specify the displayed networks:

(1) The node label represents the ICD9CM group, and the associated number, if present, is the mean number of GPP per patient;
(2) The node color represents the node strength, that is the sum of the weights from all links, and scale is shown in the picture;
(3) The node size is proportional to the number of GPP per patient referred to the specific ICD9CM group. That is, the average number of prescriptions of a specific ICD9CM group made to a patient, belonging to the considered subset, and in the studied time interval. In some figures, the exact number is given beside the node symbol (e.g., see Figure 2);
(4) The links indicate the presence of comorbidity and have a width proportional to the average number of times the corresponding ICD9CM groups have been co-prescribed per patient (link weight).

## 3. Results

### 3.1. General Population Data

The total number of analyzed GPP is 1,728,736 in the time interval 2002–2013 for 14,958 patients living in Salerno’s area. We checked whether the sample’s age distribution agrees with the age distribution of the general population living in the same area. The two groups have a clear linear dependence, as shown by the coefficient of determination measuring *r*^2^ = 0.99 for males and *r*^2^ = 0.98 for females, while a chi-square test showed *p*-values < 10^−6^ for both genders.

The first two rows of Table 1 report the number and percentage of GPP according to prescription type. The first two types, namely Drug and Laboratory Test, account for most of the prescriptions, about 89%. Among the rest, Rehabilitation and Hospital show very low values, and together account for less than 1%, while the other two, Procedures and Specialist Referral, account together for about the remaining 10%.

The third row reports the number of prescriptions per patient per year. This value was used as a guide to select the groups to study, and since a significant value was found only for Drug and Laboratory Test, the analysis has been concentrated mainly on them.

The chosen sample is consistent with the actual epidemiological status of the local population which we have checked comparing the frequencies of diseases in our sample with the official epidemiological data (source: ISTAT—Italian National Statistics Institute), for Southern Italy and Campania region [25]. Of particular interest the prevalence of diabetic patients over the total patients, reaching 7.7% in our sample compared to 6% an 5.3% of diabetic patients over the total population in Southern Italy and nationwide, respectively; the significantly higher value is probably due to the fact that ISTAT data are based on hospitals (SDO, “Scheda Dimissione Ospedaliera”, Hospital Discharge Form), while our sample includes only data from GPs.

From the epidemiological standpoint, we have selected thirteen comorbidity groups that may be referred to specific clinical sectors: i.e., CIRC, META, DIGE, MUSC, GEN, RESP, NEOP, MENT, NERV, BLD, SENS, SKIN and INFE, accounting for 81% of the total GPP. In particular, a subgroup made by META, GEN, NEOP and BLD includes the majority of GPP made by “Laboratory Test”, while for all the rest “Drug prescriptions” prevails. The excluded groups have very few GPP (PREG, CONG and NEWB), have an accidental origin (INJ), or lack connection to a specific clinical discipline (ILL and SUPP). Table 1 gives no information about possible disease linkages, and therefore, a network analysis approach must be used to bring additional insights.

### 3.2. Comorbidity Networks by Age, Gender and Prescription Type

Figure 2 shows comorbidity networks differentiated by gender (left: F) and GPP type (above: drug; below: laboratory tests).

Comparing drug vs. laboratory test prescription, we can observe very different patterns, due to the fact that laboratory test prescription is made mainly for diagnostic purposes, thus based on a hypothesis or “diagnostic question”, while the drug prescription is made based on a diagnosis, which requires an established therapy. Consequently, the study of comorbidity for epidemiological purposes should be performed based on drug co-prescription, which is medication-based data [19], to have an epidemiological framework of firm diagnoses.

Considering the firm diagnoses, on which drug prescription is based, as a reference for comparison of genders, this figure shows differentiated patterns of comorbidity, with higher prevalence for CIRC (4.15) in males and for RESP (4.1) in females. The co-prescription values are roughly the same between genders in all groups. The average number of comorbidities described by the links also shows differences: average number of GPP per patient with a diagnosis of META disease is high for both females and males (F = 9.60, M = 7.32), while other groups show differences (e.g., DIGE, higher in males; GEN, higher in females).

Considering the comorbidity pattern based on laboratory test prescription, there is an important role for META, showing the highest level for both genders, but with different networks. In fact, females reveal strong links between META and three other groups, MUSC, GEN and BLD, while males show META as strongly connected to CIRC, DIGE, GEN and NEOP. Here, in males, CIRC and DIGE, GEN and INFE are strongly connected. The lower prevalence and comorbidity of females for CIRC disease is in accordance with the estrogen protection before the menopause, still present in this age group.

### 3.3. Comorbidity Networks Evolution by Age

**Figure 3:**
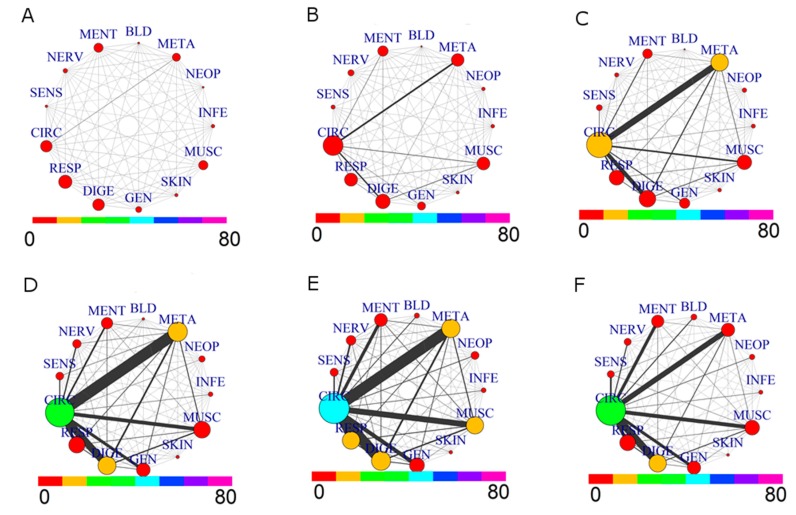
Comorbidity Networks for drug prescriptions by Age Groups. (**A**) age range: (35,45]; (**B**) age range: (45,55]; (**C**) age range: (55,65]; (**D**) age range: (65,75]; (**E**) age range: (75,85]; (**F**) age range: (85,110].

The comparison of the comorbidity patterns for the whole population (M+F) divided in six age groups from 35 to over 85 is in Figure 3.

Evolution from younger to older ages shows, as expected, a growing network of comorbidity. A complex situation appears especially for the decade 55–65: the prevalence of CIRC roughly doubles, and the same holds for META, in agreement with the increase of comorbidity between these two groups. This tendency is further evidenced in the 65–75 decade: CIRC and DIGE size increase, while the comorbidity of CIRC with META, DIGE, RESP, MUSC and GEN becomes more important. The strongest link is between CIRC and META. The next decade, 75–85, shows a similar pattern, with a significant increase of size for CIRC, RESP and MUSC diseases. The last group, 85–110, shows a different network pattern: there is a reduction in size of diseases associated to CIRC, META, MUSC and RESP, as well as for their comorbidities, with a low presence of prescription for NEOP diseases. This situation is consistent with a lower morbidity of “survivor” patients, [26] which are a small group of elderly patients who likely have not undergone severe health issues in the past.

### 3.4. Diabetes Impact on Comorbidity

**Figure 4:**
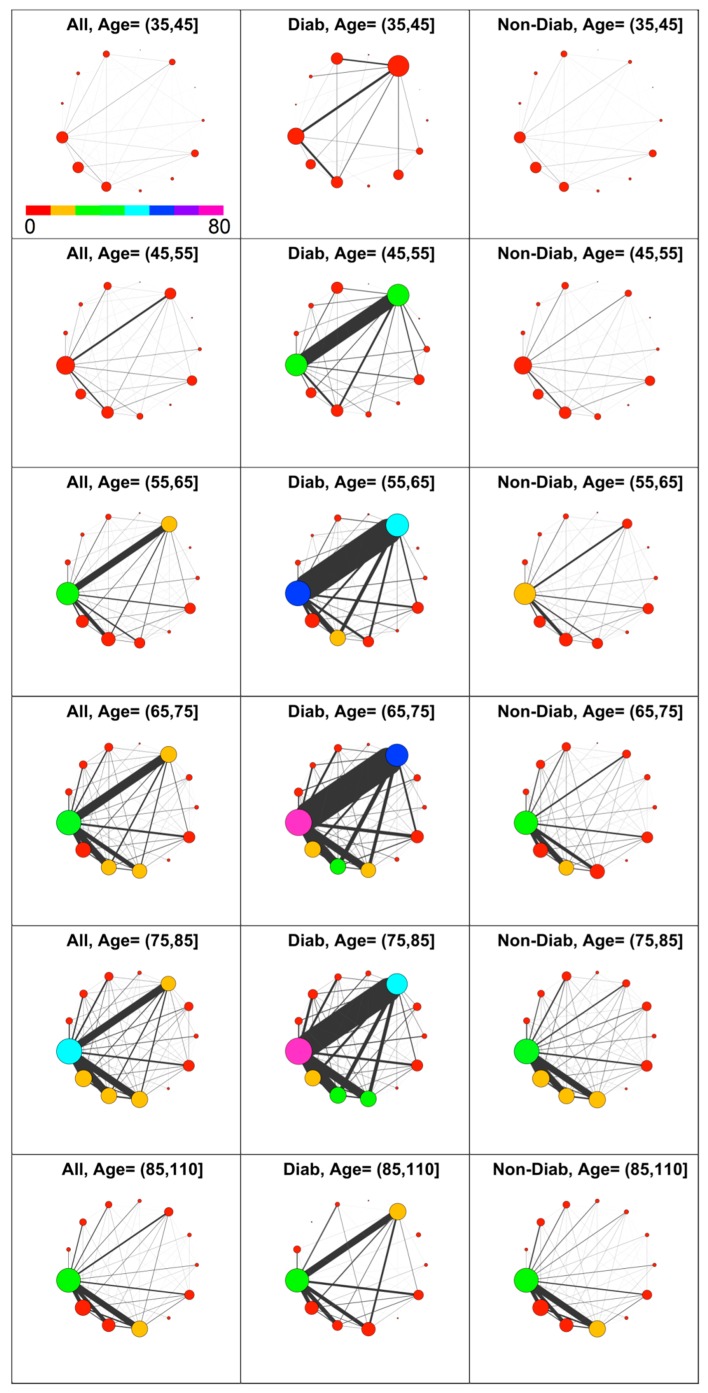
Comorbidity networks in men based on drug prescription by age group and diabetes status. Left to Right: All, Diabetic, Non-Diabetic. In the top left panel is shown the color scale that applies to all panels.

Diabetes’s impact on comorbidity was then analyzed. With reference to GPP, diabetic patients have been identified from prescribed drugs with associated ICD9 in the range 250.00–250.99. Thus, co-prescription networks have been computed for three groups, namely the patients’ population, diabetic patients only, and non-diabetic patients. The results obtained for Male patients at age decades from 35 years and more, are shown in Figure 4. Of 7246 included males, 615 have the code 250 (diagnosis of diabetes).

The diabetic patients have a higher level of comorbidity compared with the non-diabetic patients in nearly all decades.

As expected, there is a strong difference in the number of prescriptions associated to metabolic diseases (META) between diabetic and non-diabetic patients, confirming that most of diagnosed metabolic diseases are associated with diabetes. Consequently, diseases associated to CIRC have a prevalence at least twofold in diabetic patients compared with non-diabetic ones in the age interval from 45 to 85. Remarkably, for young and elderly patients, this difference is not present. Furthermore, the most evident difference between D and ND patients is the strong link between META and CIRC diseases at all ages.

This diabetes linkage shows the well-known presence of a strong adverse effect on circulation, which induces additional comorbidities with both CIRC and META. On the contrary, non-diabetic individuals show a lower circulatory morbidity, and their comorbidity with other disease, namely RESP, DIGE and GEN, rises only after the 65–75 decade. Therefore, considering the observed health demand and co-prescription volume in light of comorbidity, higher levels for diabetic individuals are revealed at nearly any age.

### 3.5. Diabetes Impact on Comorbidity by Age Group

**Figure 5:**
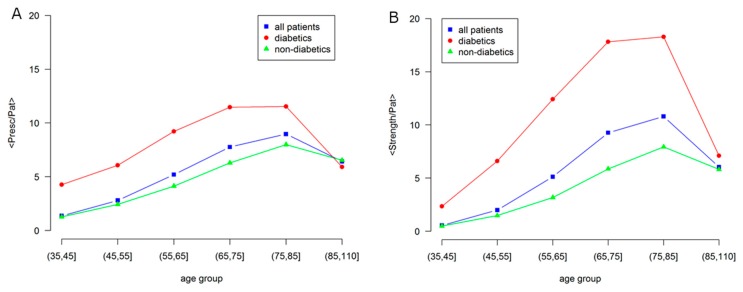
Age dependence of morbidity and comorbidity in Male patients for: all, diabetic and non-diabetic individuals. (**A**) Average number of prescriptions per patient (node size); (**B**) Network strength per patient, i.e., average number of comorbidities per patient (node color). Labels are: all patients, blue squares; diabetics, red circles; non-diabetics, green triangles.

Figure 5 represents in a more quantitative way some aspects shown by the networks in Figure 4. In particular, it shows how the number of GPP (General Practitioner Prescriptions) per patient differ according to the presence of diabetes.

The average node strength per patient is shown in the right panel, which indicates the sum of the weight of all the links of a node, a global indicator of comorbidity, as compared with the average number of prescriptions, in the left panel, as an indicator of morbidity.

A strong difference is observed between diabetic and non-diabetic individuals, confirming the effect of diabetes on the overall health status. In fact, even if there is a general increase with age, the morbidity level of the general population is similar to the one of non-diabetic individuals, while diabetic individuals show larger values of morbidity and comorbidity.

At elderly age, over 85, the morbidity levels strongly decrease and become the same. This is consistent with the low morbidity level observed in the studies of centenarians [26].

### 3.6. Comorbidity Pattern in Diabetic Patients

**Figure 6:**
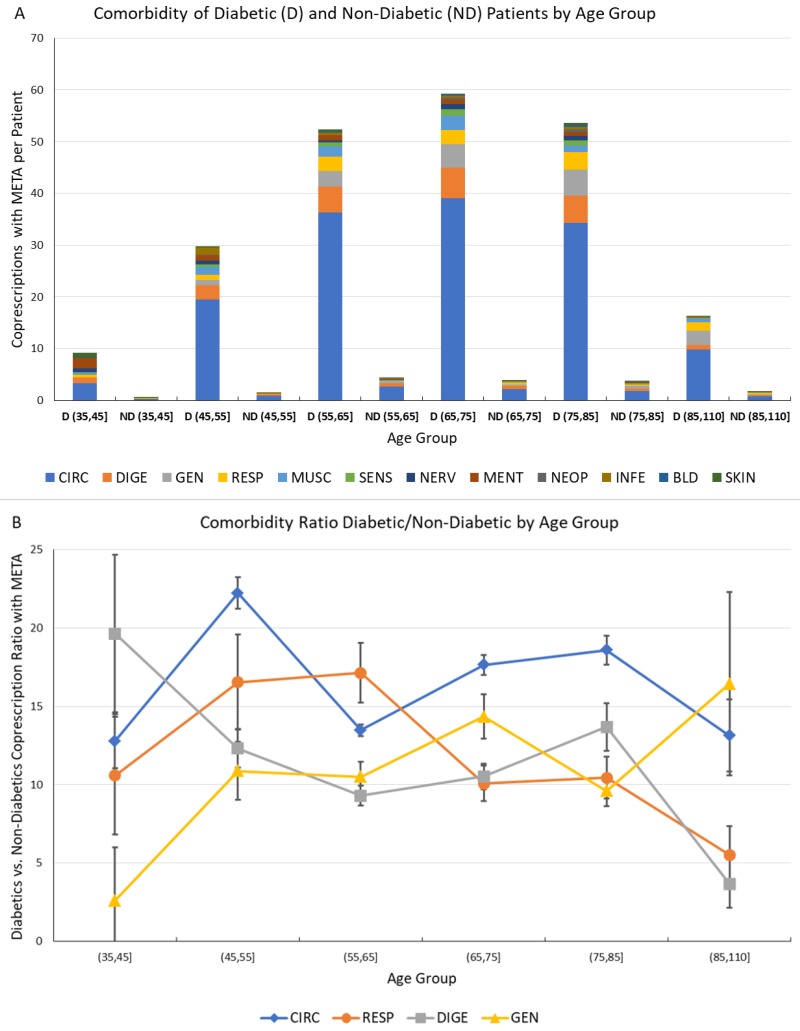
Comorbidity of Diabetic and Non-Diabetic male Patients by Age Group. (**A**) Comorbidities associated to metabolic diseases (META) for diabetic (D) and non-diabetic (ND) patients by age group. The vertical scale represents the average number of co-prescriptions with META group per patient. The colors represent the morbidity groups, as shown in the legend; (**B**) Ratio of diabetic vs. non-diabetic patient co-prescriptions associated to metabolic diseases by age groups for the most often prescribed morbidity groups (CIRC, RESP, DIGE and GEN). The error bar represents the 95% confidence interval.

Finally, the number of comorbidities associated with diabetes and included in the META group has been investigated, and is presented and commented in Figure 6.

This analysis has been performed by comparison of number of co-prescriptions per patient in the same age groups for D (diabetic) and ND subjects; to assess the significance of the computed values, the statistical error associated with the number of GPP has been computed assuming Poisson statistics, and the corresponding 95% confidence interval is shown as an error bar.

The comorbidities were much more frequent in D patients; and a very high ratio of D/ND co-prescriptions, representing the relative increase of comorbidity due to diabetes.

The ratio has shown values between 10 and 20, further confirming the heavy burden caused by diabetes both individually and systemically, and indicating the possibility of measuring and monitoring this disease burden by Network Analysis of comorbidities.

The availability of larger databases would lead to a smaller statistical error, also allowing inference on additional disease correlations.

## 4. Discussion

From GPR prevalently focused on drugs and laboratory tests (their combined frequency of prescriptions was 89.3% ± 0.5%), comorbidity networks have revealed a variety of patterns derived from patients’ measurable characteristics, especially age and gender [17,27,28].

Age is a natural cause of comorbidities, but the associations between morbidity groups in the general population have emerged due to differences in the GPP distribution. Among the involved disease groups—CIRC, META, ILL, DIGE, MUSC, GEN and RESP—those specifically associated with drug prescriptions were CIRC, DIGE, MUSC and RESP, while the rest referred to Laboratory tests.

A first important result is the identification of the role of GPR as indicators of comorbidities with reference to diabetes, and in agreement with the epidemiological status of the local population. Therefore, GPR are to be considered proxies of the general trends observed at population scale.

The proposed approach can be used to cover comorbidities at a wider breadth, given the reference population. Most comorbidity studies are focused on hospitalized patients, who are usually treated for specific and often acute disease conditions [29]. However, comorbidities in patients not undergoing hospitalization remain relatively understudied [10]. This is in part because comorbidities in general population subject to non-acute health impairment are often not clearly defined, and the diagnoses are quite differentiated, leaving space for preventive more than therapeutic actions. Hospital records are mostly reporting acute and important illnesses, while GPR refer to acute and chronic conditions not requiring hospitalization. GPR embrace a much larger percentage of population, and can provide important information on patients’ status before disease onset.

Once discharged from hospitals, patients are mostly followed by GP [30] managing a multitude of comorbidity trajectories [31]. The corresponding profiles of large portions of the population are very informative about both occasional and recurring transitions between acute and non-acute conditions. In particular, GPR allow for a rigorous consideration of emerging risk factors, such as lifestyle changes, nutrition, environmental influences. Based on such premises, preventive and intervention measures become more feasible and may induce cost savings.

Our network approach brings some of the recognized significant advantages [32], especially for inferring comorbidity conditions [20,27,33]. Seminal comorbidity studies that used network-driven analyses focused on diseases whose linkages were established through metabolic reactions [34]. We have shown that comorbidity patterns are significantly different depending on prescription types. These instruments offer a level of detail in health records that may span over general populations, whose study requires data aggregation processes and time.

Our database is currently covering patients of a single geographic area, thus with possible biases in view of generalizations. Extending the datasets capacity to include larger populations (regional or national) is expected to further validate the introduced methodology. Please note that the observed different patterns of comorbidity are informative at the epidemiological level due to the proposed population segmentation, and are useful also for policy makers interested in the economic impact of comorbidity in view of planning prevention programs.

It is necessary to extend the study of comorbidity conditions with any inference tool, including networks, due to the epidemiological relevance of addressing both comorbidity incidence and prevalence in stratified populations and due the need of improving the quality of assessment of their economic impact by the policymakers who design prevention programs.

The significance of network inference is destined to increase with more data available at a systems scale. This would eventually translate into a more complete definition of comorbidities during diagnostic and therapeutic phases of the healthcare process. Additional statistical power will come from longitudinal databases, already available to health organizations, and from the investigation of the temporal evolution of comorbidity, i.e., trajectories. Novel correlative patterns between different GPR will emerge from the consideration of factors such as geographic setting and lifestyle of general populations.

The information provided by the GPR dataset based on the type of intervention prescribed (Drug, Laboratory Test, etc.) suggests the need of exploring co-prescription networks with flexible tools to handle heterogeneous features (see for instance multilayer network, especially including the temporal dimension [35]). As network layers may be induced by specific GPR, the interconnected layers would indicate patient’s prescriptions from different types of interventions. An open question is how to measure (i.e., assigning weight to links) the simultaneous prescriptions according to their different frequencies across patients. Similarly open is the problem of assessing how connectivity patterns determining network dynamics could be affected by constraints referred to both normative and medical aspects.

Currently, our concluding remarks are:

(1) GPR represent a comprehensive source of information on population health to study comorbidities associated with non-acute pathological conditions observed in non-hospitalized general populations;
(2) Network analysis is an instrument to measure associations between morbidities and their dependence on health determinants;
(3) Extracting information on general population comorbidities from GPR may impact both clinical practice and health system policy making;
(4) The proposed methodology is scalable to even larger datasets and generalizable to diversified contexts influencing health through factors such as geo-localization, lifestyle, nutrition and their temporal evolution.

## Figures and Tables

**Table 1:** Distribution of General Practitioner Prescriptions (GPP) among types and ICD9CM groups (EXT—external causes of jnjury prescription group omitted).

**GPP Type**	**All**	**Drug**	**Laboratory Test**	**Procedures**	**Rehab**	**Referral**	**Hospital**
Prescriptions	1,728,736	897,329	647,023	105,126	8388	65,734	5136
% of total	100.0%	51.9%	37.4%	6.1%	0.5%	3.8%	0.3%
Prescriptions per patient per year	10.51	5.45	3.93	0.64	0.05	0.40	0.03
**GPP Type (row)**	**All**	**Drug %**	**Laboratory Test %**	**Procedures %**	**Rehab %**	**Referral %**	**Hospital %**
**ICD9 Group (column)**							
CIRC—circulatory	451,765	69.2%	25.4%	3.5%	0.0%	1.8%	0.2%
META—metabolic	231,541	37.6%	56.6%	2.5%	0.0%	3.3%	0.1%
ILL—ill defined	219,664	26.7%	56.4%	10.7%	1.0%	4.8%	0.5%
DIGE—digestive	133,736	72.6%	20.4%	4.8%	0.0%	1.9%	0.3%
MUSC—muscular	122,718	57.4%	22.2%	11.8%	3.7%	4.6%	0.3%
GEN—genitourinary	112,029	37.6%	51.6%	6.7%	0.0%	3.8%	0.3%
RESP—respiratory	110,587	87.1%	6.9%	3.2%	0.1%	2.5%	0.2%
SUPP—supplementary	75,458	13.0%	76.9%	6.5%	0.1%	3.3%	0.3%
NEOP—neoplastic	72,708	15.2%	67.7%	12.2%	0.0%	4.0%	0.9%
MENT—mental	45,328	89.5%	4.9%	0.7%	0.0%	4.8%	0.1%
NERV—nervous	28,969	69.0%	16.9%	7.3%	0.4%	5.8%	0.6%
BLD—blood	28,406	20.5%	75.6%	1.3%	0.0%	2.4%	0.2%
SENS—sensory	26,879	57.1%	5.3%	14.0%	0.0%	22.0%	1.5%
SKIN	22,250	44.3%	36.2%	3.7%	0.1%	15.4%	0.4%
INFE—infectious	21,803	49.5%	33.1%	11.5%	0.1%	5.6%	0.2%
INJ—injuries	20,342	40.1%	14.7%	21.7%	5.3%	17.5%	0.7%
PREG—pregnancy	2748	41.9%	48.2%	4.4%	0.0%	3.2%	2.2%
CONG—congenital	1401	24.1%	42.1%	18.6%	1.1%	10.5%	3.5%
NEWB—newborn	404	66.6%	21.0%	2.0%	0.0%	8.9%	1.5%

## Supplementary Materials

The following is available online at http://www.mdpi.com/2077-0383/7/8/184/s1, (Comorbidity Network Analysis Supplementary Digital Content).
