# Supplementary material for: General Practitioners Records Are Epidemiological Predictors of Comorbidities: An Analytical Cross-Sectional 10-Year Retrospective Study
Source: J Clin Med. 2018 Jul 27;7(8):184. doi: 10.3390/jcm7080184 (PMC6111778; doi:10.3390/jcm7080184)
Supplement: Supplementary file 1 [file jcm-07-00184-s001.pdf]

Comorbidity Network Analysis - Supplemental Digital Content

Table S1: ICD9 Morbidity Groups with details and examples

From	To	Acronym	Description	Sub-groups	Details and Examples
**001**	**139**	**INFE**	**Infectious and parasitic diseases**	**16**	**Classification by district and/or biological agent.<br>001-009 = Intestinal infections. 001 = Cholera. 006.3 = Amoebic liver abscess.<br>020-027 = Bacterial Zoonoses. 022 = Anthrax.<br>042-044 = HIV infection.<br>110-118 = Mycoses. 112 = Candidiasis. 112.1 = Moniliasis, vulva/vagina.**
**140**	**239**	**NEOP**	**Neoplasms**	**13**	**Classification by anatomic district and/or biological features.<br>150-159 = Digestive organs. 153 = Colon malignancy. 153.3 = Sigmoid colon.<br>170-175 = Malignant neoplasms of bone, connective tissue, skin and breast.<br>210-229 = Benigne neoplasms. 218 = Uterine leiomyoma.**
**240**	**279**	**META**	**Endocrine, nutritional and metabolic diseases, and immunity disorders**	**4**	**Classification by gland, nutrient or metabolic/immunologic function.<br>240-246 = Disorders of the thyroid gland. 244 = Hypothyroidism.<br>249-259 = Disorders of the thyroid gland. 250 = Diabetes mellitus.<br>267 = Ascorbic acid deficiency.<br>272.0 = Pure hypercholesterolemia.<br>277.0 = Cystic fibrosis.<br>278 = Obesity.<br>279.00 = Hypogammaglobulinemia.**
**280**	**289**	**BLD**	**Diseases of the blood and blood forming organs**	**3**	**Classification by blood cell type.<br>280-285 = Anemia. 283 = Acquired hemolytic anemia.<br>286-287 = Coagulation/hemorrhagic.<br>288-289 = Other. 288.51 = Lymphocitopenia.**
**290**	**319**	**MENT**	**Mental disorders**	**3**	**Classification by type.<br>290 – 299 = Psychosis. 295 = Schizophrenic disorders.<br>300-316 = Neuroses and personality disorders. 300.8 = Somatoform disorders.<br>317-319 = Mental retardation.**
**320**	**359**	**NERV**	**Diseases of the nervous system**	**6**	**Classification by type and/or anatomical district.<br>320-327 = Inflammatory diseases, central. 323 = Encephalitis.<br>338.2 = Chronic pain.<br>350-359 = Disorders of the peripheral system. 354.0 = Carpal tunnel syndrome.**

From	To	Acronym	Description	Sub-groups	Details and Examples
360	389	SENS	Diseases of the sense organs	2	Classification by organ.<br>360-379 = Eye. 362.0 = Diabetic retinopathy. 366 = Cataract.<br>380-389 = Ear. 386.01 Meniere's disease. 389 = Deafness.
390	459	CIRC	Diseases of the circulatory system	9	Classification by type and/or anatomical district.<br>393-398 = Chronic rheumatic heart disease.<br>401-405 = Hypertensive disease. 402 = Hypertensive heart disease.<br>410-414 = Ischemic heart disease. 414.0 = Coronary atherosclerosis.<br>430-438 = Cerebrovascular disease. 437.2 = Hypertensive encephalopathy.<br>451-459 = Disease of veins and lymphatics. 454 = Varicose veins, lower limb.
460	519	RESP	Diseases of the respiratory system	6	Classification by type.<br>460-519 = Acute respiratory infections. 466.0 = Bronchitis, acute.<br>480-488 = Pneumonia and Influenza. 488.1 = H1N1 Influenza.<br>490-496 = Chronic obstructive pulmonary disease.<br>510-519 = Other diseases. 516.3 = Idiopathic fibrosing alveolitis.
520	579	DIGE	Diseases of the digestive system	8	Classification by type and/or anatomical district.<br>520-529 = Diseases of the oral cavity. 521.0 = Dental caries.<br>531 = Gastric ulcera.<br>550 = Inguinal hernia.<br>555-558 = Noninfectious enteritis and colitis. 555.0 = Crohn, small intestine.<br>571.0 = Fatty liver, alcoholic.
580	629	GEN	Diseases of the genitourinary system	5	Classification by type and/or anatomical district.<br>580-589 = Nephritis and nephrosis. 585 = Chronic renal failure.<br>595 = Cystitis. 598 = Urethral stricture.<br>600-608 = Male genital organs. 601 = Inflammatory disease of prostate.<br>610-612 = Disorders of breast.<br>617-629 = Female genital tract. 622.1 = Dysplasia of uterine cervix.
630	679	PREG	Complications of pregnancy, childbirth, and the puerperium	7	Classification by type.<br>634-639 = Pregnancy with abortive outcome.<br>650-659 = Normal delivery and other indications. 651 = Multiple gestation.<br>660-669 = Complications of labor and delivery. 663.1 = Cord around neck.
680	709	SKIN	Diseases of the skin and subcutaneous tissue	3	Classification by type.<br>680-686 = Infections. 682.1 = cellulitis/abscess of the neck.<br>690-698 = Other inflammatory conditions. 692.71 = Sunburn.

From	To	Acronym	Description	Sub-groups	Details and Examples
710	739	MUSC	Diseases of the musculoskeletal system and connective tissue	5	Classification by type and/or anatomical district.<br>710-719 = Arthropaties. 710.4 = Polymyositis.<br>720-724 = Dorsopathies. 722.52 = Degenerative disc disease, lumbar.<br>725-729 = Rheumatism, excluding the back. 729.1 = Fibromyositis.<br>730-739 = Osteopathies. 733.0 = Osteoporosis.
740	759	CONG	Congenital anomalies	11	Classification by anatomical district.<br>742.1 = Microcephalus.<br>745-747 = Circulatory system. 746.5 = Congenital mitral stenosis.<br>753.1 = Cystic kidney disease.
760	779	NEWB	Certain conditions originating in the perinatal period	2	Classification by type and/or anatomical district.<br>767 = Birth trauma.<br>771.0 = Congenital rubella.
780	799	ILL	Symptoms, signs, and ill-defined conditions	3	Classification by type and/or anatomical district.<br>780-789 = Symptoms. 780.8 = Sweating, excessive. 780.93 = Memory loss.<br>790-796 = Nonspecific abnormal findings. 791.0 = Proteinuria.
800	999	INJ	Injury and poisoning	24	Classification by anatomical district.<br>801 = Fracture of the base of skull.<br>812 = Fracture of humerus.<br>865 = Injury to spleen.
E00	E99	EXT	External causes of injury	26	Classification by type, namely the origin of injury.<br>E800-E807 = Railway accident.<br>E810-E819 = Motor vehicle traffic accidents. E814 = Collision with pedestrian.<br>E840-E845 = Air and space transport accidents.<br>E900-E909 = Natural factors. E906.0 = Dog bite.<br>E950-E959 = Suicide and self-inflicted injury.<br>E979 = Terrorism.
V00	V99	SUPP	Supplemental classification	17	This group includes factors influencing health status and contact with health services, classified by type.<br>V07-V09 = Persons with need for isolation and prophylactic measures.<br>V50-V59 = Persons encountering health services for specific procedures

Prescription rules for General Practitioners in Italy

In Italy the GPs are the main interface between the patient and the National Health Service (NHS), excluding emergency requests.

A GP is associated to each Italian citizen or resident, as reference for any health issue: after each visit the GP can prescribe drug therapy, laboratory testing, imaging, specialist referral, rehabilitation, and hospitalization. All the prescriptions are recorded for administrative purposes of the NHS.

The GPs administrative prescription data consists of anonymized patient ID, age, sex, date of prescription, prescription type, prescription code, diagnosis code.

The prescription code refers to the prescribed item, and is contained into a document published and periodically updated by the Italian Government ("Nomenclatore Tariffario"). In case of a drug prescription, it corresponds to the Anatomical Therapeutic Chemical (ATC) classification (World Health Organization 2015).

The diagnosis code associates a diagnosis to each prescription, and follows the International Classification of Diseases, Ninth Revision, Clinical Modification (ICD9CM) (World Health Organization 2010). The data accuracy is automatically verified by the GP clinical records software.

The ICD9CM is the standard diagnostic tool for epidemiology, health management and clinical purposes used by the Italian National Health System.

It has the general form 'xxx.yy', where the 'xxx' segment is the general disease and the 'yy' segment is a specific occurrence, e.g. 250 is the code for "Diabetes", and 250.91 is the code for "Diabetes Type 1 (juvenile) with unspecified complication, not stated as uncontrolled".

Our approach then groups the diagnostic codes into specific epidemiological areas corresponding to the 20 main groups of the ICD9CM classification, listed in the present document, Table S1.

References

World Health Organization. (2010). "International Classification of Diseases (ICD)." from http://www.who.int/classifications/icd/en/.

World Health Organization (2015). Guidelines for ATC classification and DDD assignment 2015. Oslo, WHO Collaborating Centre for Drug Statistics Methodology.
